# Editorial: Foods and Macronutrients in NAFLD: Associations, Effects and Mechanisms

**DOI:** 10.3389/fnut.2021.665436

**Published:** 2021-03-23

**Authors:** Fredrik Rosqvist, Leanne Hodson, Barbara A. Fielding

**Affiliations:** ^1^Department of Public Health and Caring Sciences, Clinical Nutrition and Metabolism, Uppsala University, Uppsala, Sweden; ^2^Oxford Center for Diabetes, Endocrinology and Metabolism, Churchill Hospital, University of Oxford, Oxford, United Kingdom; ^3^Oxford NIHR Biomedical Research Center, Churchill Hospital, Oxford, United Kingdom; ^4^Faculty of Health and Medical Sciences, University of Surrey, Guildford, United Kingdom

**Keywords:** NAFLD, foods, dietary macronutrients, dietary quality, prevention, management, treatment

Non-alcoholic fatty liver disease (NAFLD), characterized by an excessive accumulation of fat in the liver, is associated with type 2 diabetes (1), cardiovascular disease (2), and mortality (3). NAFLD is highly prevalent (4) and thus constitutes an important burden to public health. Although being overweight is an important driver of NAFLD, dietary composition may influence liver fat accumulation independent of changes in body weight (5). However, more research is needed to identify dietary factors that may influence NAFLD and which mechanisms are at play. Thus, the goal of this Research Topic was to further our understanding of the interactions between dietary intake and NAFLD development to inform the development of dietary strategies for the prevention and treatment of NAFLD.

There has been much focus on the importance of dietary fats and carbohydrates, and their subtypes, for liver fat accumulation. Hydes et al. performed a narrative literature review in this Research Topic, with a primary focus on overfeeding studies, and discuss potential mechanisms. The authors state that overfeeding with simple sugars leads to increased liver fat, through increased *de novo* lipogenesis, but indicated that additional studies are needed to investigate whether this is independent of weight gain. The authors further state that overfeeding of fat also leads to increased liver fat but that the type of fat is a clear determinant of the effect. Saturated fat appears particularly harmful, potentially mediated through increased lipolysis and ceramides, whereas polyunsaturated fat are protective. The authors continue with a discussion on potential mediators, such as genetics, and call for additional studies in this area. The importance of physical activity for counteracting the effects of overfeeding is highlighted and the authors conclude that prevention and disease modification through dietary recommendations have huge potential to benefit patients with or at risk for NAFLD.

Carbohydrate quality may be more important than carbohydrate quantity regarding risk for NAFLD, and metabolic health in general. One measure of dietary carbohydrate quality is intake of fiber. Zhao et al. investigates the cross-sectional relationship between dietary fiber from different sources and risk of NAFLD in a large cohort of US adults. After adjusting for multiple confounders, the authors demonstrate striking inverse relationships between dietary fiber and risk of NAFLD, as assessed by a fatty liver index. Odds ratios of NAFLD for the highest vs. lowest quartile of fiber ranged between 0.12 and 0.42 for total, cereal, fruit, and vegetable fiber. These inverse associations remained in analyses stratified by sex and age. Dose-response analysis indicated linear relationships between cereal, fruit, and vegetable fiber and odds ratio of NAFLD whereas the relationship with total fiber intake was non-linear.

Similar to carbohydrates, the quality of dietary fat appears more important than its quantity. As shown in the review by Hydes et al., excessive intake of saturated fat promotes liver fat accumulation at the group level. However, this response may differ between individuals. Rosqvist et al. et al. performed a pooled analysis on two previously conducted randomized overfeeding trials with the aim to identify predictors of liver fat accumulation in response to an increased intake of saturated fat. The authors demonstrated that there exists high inter-individual variation in liver fat accumulation during a hypercaloric diet rich in saturated fat that could not be explained by changes in body weight. Positive predictors included amount of visceral adipose tissue, liver fat content, insulin resistance, and some fatty acids in the circulation and adipose tissue whereas adiponectin and the odd-chain fatty acid 17:0 in adipose tissue were the only negative predictors. Based on a linear regression analysis, the authors demonstrated that 81% of the variation in liver fat change could be predicted by eight variables assessed at baseline.

Much effort has been put on understanding the roles of individual nutrients (e.g. saturated fat, fructose). However, in real life, dietary habits are complex and the effects of single nutrients may be modified by the overall dietary pattern. Xia et al. address this complexity by using network science to construct dietary networks in a Chinese case-control study in subjects with newly diagnosed NAFLD and matched controls. Although cases and controls had similar absolute intakes of many individual food groups, the authors were able to demonstrate different overall dietary structures between groups and suggested that a diverse diet focusing on whole-grains, tubers, and vegetables may be preventive for NAFLD. The authors conclude that how foods are consumed, in addition to the absolute intake, could be important in determining the occurrence of NAFLD and that more focus is needed on the whole dietary structure.

Dietary strategies to prevent NAFLD are highly needed, but effective dietary strategies to manage patients already with NAFLD should not be forgotten. Patients with NAFLD have an increased risk of developing cardiovascular disease, a risk that may partly be mitigated by improving the blood lipid profile. Dietary choices may have profound effects on plasma lipoproteins however dietary factors may have less pronounced effects in subjects with obesity and it is unclear if the presence of NAFLD modifies these effects. Thus, Rosqvist et al. systematically evaluated the literature for the effects of foods and dietary patterns on blood lipids in adults with NAFLD. Based on limited evidence, the authors found that no foods or dietary patterns modified blood lipids with “High quality” evidence. However, with “Moderate quality” evidence, a healthy dietary pattern was found to reduce triglycerides compared with standard care and probiotic yogurt may decrease plasma total cholesterol compared with conventional yogurt. The authors concludes that their results, overall, are mostly in line with current guidelines for the treatment of dyslipidemia or prevention of cardiovascular disease in other populations but that it is not possible to determine which foods significantly modify blood lipids in adults with NAFLD based on the available evidence.

Although the majority of research focuses on adults, NAFLD has become the leading cause of liver disease also in the pediatric population. Suboptimal maternal nutrition may play an important role in the pathogenesis of pediatric NAFLD but mechanisms are unclear. Using a rat model in combination with an *in vitro* cellular model of HepG2 cells, Cao et al.. explored the effect of a maternal high-fat diet in the pathogenesis of neonatal NAFLD and its underlying mechanisms. As previously demonstrated, maternal high-fat diet (vs. normal diet) led to increased liver fat in the neonates. The authors further demonstrated that a maternal high-fat diet leads to intrauterine inflammation and upregulation of the lipogenic enzyme SCD in the livers of neonates. Experiments in HepG2 cells showed that treatment with the inflammatory cytokine IL6 led to increased expression SCD and that this resulted in increased intracellular triglyceride content. The authors thus suggested that treatment targeting SCD and/or inflammation could be potential options for management of NAFLD.

## Author Contributions

FR wrote the first draft. LH and BF critically revised the draft. All authors contributed to the article and approved the submitted version.

## Conflict of Interest

The authors declare that the research was conducted in the absence of any commercial or financial relationships that could be construed as a potential conflict of interest.
